# Molecular Dynamic and Dissipative Particle Dynamic Simulation on the Miscibility of NR/CR Blends

**DOI:** 10.3390/polym15040856

**Published:** 2023-02-09

**Authors:** Yanbin Ma, Xiaoqin Yuan, Ruifeng Jiang, Jianhe Liao, Rentong Yu, Yongping Chen, Lusheng Liao

**Affiliations:** 1 School of Life Sciences, Hainan University, Haikou 570228, China; 2 School of Materials Science and Engineering, Hainan University, Haikou 570228, China; 3 Hainan Provincial Key Laboratory of Natural Rubber Processing, Agricultural Products Processing Research Institute of Chinese Academy of Tropical Agricultural Sciences, Zhanjiang 524001, China

**Keywords:** natural rubber, rubber blending, compatibility, molecular dynamics simulation, dissipative particle dynamics simulation

## Abstract

Natural rubber (NR) exhibits good elasticity, flexural resistance, wear resistance, and excellent mechanical properties, and it has been widely used in aerospace, transportation, medical, and health fields. For NR, however, the resistance to thermal-oxidation and ozone aging is fairly poor. Although aging properties of NR can be significantly improved with the incorporation of chloroprene rubber (CR) according to some references, the miscibility between NR and CR, the morphologies of the binary blends, and so on are revealed ambiguously. In this work, molecular dynamics simulation (MD) and dissipative particle dynamics (DPD) simulation were carried out to predict the compatibility between natural rubber and chloroprene rubber in view of Flory–Huggins parameters. The morphologies of the blends were obtained with the use of the DPD method. The simulation results were furtherly examined by means of Fourier transform infrared spectroscopy (FT-IR) and dynamic mechanical analysis (DMA). It was found that the miscibility between NR and CR is poor. Nevertheless, the miscibility could be improved when the content of CR is 50% or 90%. In addition, spinodal decomposition with a critical temperature of 390 K would take place according to the phase diagram. Microphase structure such as spherical, lamellar, and bicontinuous phases can be found with different contents of CR in the blends with the results of morphologies analysis.

## 1. Introduction

With the rapid development of society, the performance requirements of polymers have been more and more stringent. To meet this need, enormous effects have been devoted for the purpose of polymer modification. Of them, polymer blending has become an effective and cost-saving methodology [1,2]. Natural rubber (NR), a natural polymer obtained from *Hevea brasiliensis*, is conspicuous for its excellent mechanical properties and high insulation. It is widely recognized that dynamic flexibility originated from the double-bond structure endows NR marvelous toughness. The unsaturated structure also affords NR versatile chemical modification strategies. On the other hand, NR is very susceptible to oxidation due to the highly unsaturated structure, which results in poor thermal-oxidative aging resistance and ozone resistance [3,4,5,6]. Nevertheless, not all polymers containing unsaturated repeating units are prone to being oxidized. In comparison with natural rubber, the chlorine in chloroprene rubber (CR) reduces the reactivity of double bonds and thus improves its chemical resistance. Accordingly, CR displays outstanding resistance to thermal-oxidation aging and ozone cracking. By blending NR with CR, a compound (NR/CR) with good corrosion resistance and weather resistance can be anticipated, which can be used in the manufacture of wires, cables, sealing gaskets, and auto parts [7,8,9,10]. Huang [11] et al. developed a NR/CR blending technology and investigated the effect of mass ratio between NR and CR on the mechanical properties of blends. They found that NR/CR blends exhibited better mechanical properties than NR. With the increase of CR content, solvent resistance and flame resistance can both be improved. On the contrary, flame retardancy and weather resistance would be weakened with high incorporation of NR in the blends. Kongvasana et al. [12] investigated the properties of natural rubber/chloroprene rubber (NR/CR) blends under thermal and thermal-oil aging. The results showed that tensile strength of the NR/CR vulcanizates was slightly reduced after thermal aging especially at high NR content, more extreme reduction being found by thermal-oil aging. Malomo et al. [13] explored the physico-mechanical, solubility, and thermodynamics of NR/CR blends; the results showed that the blends’ composition played a significant role in organic solvents uptake, and physico-mechanical properties obtained were impaired at high neoprene content. Nevertheless, the relationship between properties and morphologies were not clarified in detail.

It is well known that properties of polymer materials are considerably governed by their morphology. As for NR and CR, the former is a typical nonpolar polymer while the other is a polymer with characteristic of polarity. The compatibility between them plays an important role in the morphology, phase structure, and properties of the blends’ inevitably. Homogeneous phase structure due to good compatibility and macroscopic phase separation owing to complete incompatibility are not desirable for polymer blending [14]. In contrast, performance can often be enhanced by reason of multiphase structure of materials. Thus, it is very necessary to investigate the miscibility and morphology of polymer blends. The miscibility of polymer blends can be analyzed by glass transition temperature, which can be measured by means of dynamic thermomechanical analysis (DMA) and differential scanning calorimetry (DSC), and also can be illustrated through morphology detected by scanning electron microscopy (SEM), transmission electron microscopy (TEM), etc. To afford this, expenditure of resources and research time should be charged in plenty. Even so, the results obtained from traditional characterization and measurement technologies could be ambiguous. For example, the glass transition temperature of NR tested by DSC could be hard to be determined accurately by reason of entropy relaxation. In addition, the craft of sample preparation may have an effect on the results. It is especially critical for cryoultramicrotomy of elastomers. In this sense, the development of polymer materials is still largely empirical, and the relationship between properties and structure cannot be achieved so far.

Fortunately, with the development of computer technology, molecular simulation has been widely used in various materials fields and is favored by scientific researchers. Molecular simulation can not only predict information of structure together with properties of polymers, but also provide explanations of experimental observations at different length and time scales. Many computational methods have been applied for exploration of polymer materials, which should be well chosen according to object scale. For example, molecular dynamics or Monte Carlo methods have often been adopted to investigate polymers at molecular scale. Dissipative particle dynamics (DPD), Brownian dynamics, dynamic density functional theory method, and so on have been widely used to probe polymers at mesoscale scale. In addition, some methods such as micromechanics have been employed at the level of macroscale. To achieve materials for meeting the needs of engineering, NR/CR blends were furtherly modified with other components. It is worthy of expectation that morphologies of the blends could be explored beforehand in depth with the help of molecular simulation to turn the properties desired. With the aid of molecular simulation, morphological evolution, phase separation, and so on, it can be elucidated in polymer blending systems. Structure and properties of the blends can also be predicted with the results of molecular and atomic scales analysis [15,16].

Although molecular simulations are widely used in polymer blending systems, most of them have focused on plastic blending systems or other composite material systems, but there are not many molecular simulation studies on rubber blend systems. Yang Jinmeng et al. [17] used molecular simulation to study the compatibility and glass transition temperature of an NR/BR blending system. The results showed that the blending system exhibited good compatibility when the mass fraction of BR was less than 20%. When the mass fraction of BR was more than 30%, phase separation occurred, and the compatibility became poor. With the increase of BR mass fraction, the glass transition temperature of the blending system decreased, and the property of cold resistance was improved. Jiang et al. [18] studied the mechanical properties of natural rubber/gutta-percha (TPI) by molecular simulation. The results showed that the NR/TPI blending system demonstrated higher strength and elasticity than pure rubber, but the modulus and stiffness were decreased. Ryu et al. [19] developed an optimization equation that could predict the glass transition temperature of various rubber blending systems by using the molecular simulation method, which was important in guiding rational design of tires. For all that, there are still only a small number of molecular simulation research studies carried out on elastomer or rubber, and most of the works were concerned with properties other than structure.

Recently, functional polymer materials in constitution of NR and CR have been fabricated and investigated. For instance, sensing characteristics of conductive chloroprene rubber/natural rubber composites were examined in consideration of a coblending process [20]. The morphologies of the blends would have a remarkable effect on the properties. As a matter of fact, the methodology of tuning properties can be improved by the use of molecular simulation based on the relationship between properties and structure.

In this work, we investigated the compatibility between NR and CR by calculating the solubility parameters and the Flory–Huggins parameter using the method of molecular dynamics. Then we constructed the phase diagram of the NR/CR blends. The morphologies of the blends were visualized by using the DPD method. In addition, FT-IR and DMA experiments were carried out to support the results of molecular simulation.

## 2. Theory and Method

### 2.1. Classic Molecular Dynamics Simulation

Materials studio (MS) is a commercial molecular simulation software developed by Accelrys Company, which includes functions of modeling, structure optimization, performance calculation, and so on. The software contains many functional modules to perform molecular dynamics, molecular mechanics, quantum mechanics, and other computational simulation methods, which can realize the simulation of materials from microscopic to mesoscopic scales. At present, Materials studio is widely used in petrochemical, food and pharmaceutical, biochemistry, and other fields, and is often used in the research of polymer material properties, surface chemistry, surface catalysis, and other issues.

Molecular dynamics simulation (MD) is based on Newtonian mechanics, simulates the motion of particles by applying forces and torques, extracts relevant information from the system, and analyzes the performance of the system. The motion law of particles in the system follows Newton’s equation of motion:

(1) $$F_{i}=-\nabla_{i}U=-\left(i\frac{\partial }{\partial x_{i}}+i\frac{\partial }{\partial_{y_{j}}}+i\frac{\partial }{\partial_{z_{k}}}\right)U$$

The acceleration of particle *i* is:

(2) $$a=\frac{F_{x_{i}}}{m_{i}}$$

By integrating Lagrange’s equation of motion over time, the acceleration, velocity, and position of particle *i* after time *t* can be obtained:

(3) $$\frac{d^{2}}{dt^{2}}r_{i}=\frac{d}{dt}v_{i}=a_{i}$$

(4) $$v_{i}=v_{i}^{0}+a_{i}t$$

(5) $$r_{i}=r_{i}^{0}+v_{i}^{0}t+\frac{1}{2}a_{i}t^{2}$$

In the formula, $F_{i},-\nabla_{i}U,\;U$ are the force, potential energy function gradient, and potential energy function that particle *i* is subjected to, respectively; $a_{i},\;v_{i},\;t,\;m_{i},\;\mathrm{and}\;r_{i}$ are the acceleration, velocity, time, mass, and position of particle *i*, respectively; and the superscript “0” is the initial value of each physical parameter.

MD simulations themselves have become more powerful and accessible over the past few years [21]. Ndoro et al. [22,23] used MD simulations at isothermal isobaric (NPT) ensemble, i.e., atmospheric pressure and temperature above the glass transition temperature of PS, to study the PS structure within a nanocomposite that consisted of spherical silica nanoparticles embedded in an atactic PS matrix. Brown et al. [24] predicted the interfacial structure and unperturbed polymer chains in a nanocomposite with silica nanoparticles dispersed in the polyethylene (PE) matrix. Eslami et al. [25] investigated the structure and interface of bare or grafted silica nanoparticles of various diameters in an oligomeric poly methyl methacrylate (PMMA) matrix.

In this study, the Materials Visualizer module of Materials Studio (MS) software was used to construct the NR/CR blends with different mass ratios, and molecular dynamics methods were used to optimize the model and calculate the performance of blends; the miscibility of the NR/CR blends was analyzed with Flory–Huggins interaction parameters and phase diagrams.

**Figure 1:**
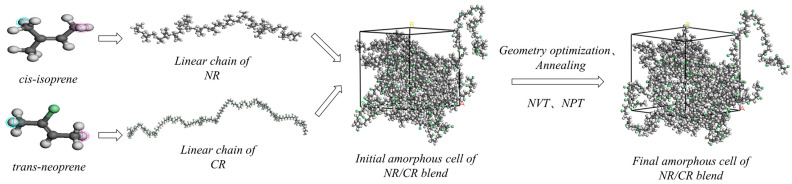
Modeling process of the NR/CR blends. (White corresponds to hydrogen atoms, gray corresponds to carbon atoms, and light green corresponds to chlorine atoms. A, B, and C mean the end-point positions of the modeling box along the *x*, *y*, and *z* axis, respectively.)

The modeling process of the NR/CR blends is depicted in Figure 1, and the initial model-building parameters of the NR/CR blends are shown in Table 1. Firstly, the NR and CR monomers were imported using the MS Import tool, and then the NR and CR single-molecule chains were constructed using the Build polymer tool to optimize the geometry of the single chain. After that, Amorphous molecular models of the NR/CR blends with mass ratios of 90/10, 70/30, 50/50, 30/70, and 10/90 were constructed using the Amorphous cell module. The final NR/CR blending model was obtained by using Forcite’s Geomestry optimization module of the Materials Studio software.

The models were furtherly annealed with a temperature gradient of 50 K, rising from 300 K to 500 K, and then decreased from 500 K to 300 K. Five cycles were performed to eliminate unreasonable configurations. The annealed models were then balanced for 100 ps of NVT together with 200 ps of NPT. Every trajectory was saved per ps. The last 50 ps trajectories were used for performance analysis. The whole molecular dynamics simulation process adopted the COMPASS force field [26], Nosé–Hoover thermostat method [27], and Berendsen pressure control method [28]. The calculation was executed on electrostatic force and van der Waals force. The nonbonded cutoff distance of the model was set to 0.95 nm. The spline width and buffer width were set to 0.1 nm and 0.05 nm, respectively. The time step was 1 *f*s.

### 2.2. Dissipative Particle Dynamics Simulation

Dissipative particle dynamics (DPD) is a simulation method at the mesoscopic scale proposed by Hoogerbrugge and Koelman in 1992 [29]. Unlike classic molecular dynamics simulation, DPD simulation does not consider the behavioral details of atoms. The particles of the system are not isolated atoms or molecules, but “beads” composed of a certain number of atoms, molecules, or statistical fragments. DPD simulation is based on these bead-spring models, linking molecular dynamics and thermodynamics. The process of phase separation can be simulated, and the evolution of the mesoscopic appearance will be acquired [30,31,32]. DPD simulation can also be used to study morphology and properties of polymer–nanoparticle mixtures, polymer blends, longer time scale processes, hydrodynamical phenomena, and so on [33,34,35,36].

The starting point of the dissipative particle dynamics simulation formula is the integral Newton equation, and the dynamic behavior between the beads can be described by the Newtonian equation of motion:

(6) $$\frac{dr_{i}}{dt}=v_{i},\;\;\;m_{i}\frac{dv_{i}}{dt}={ƒ}_{i}$$

where $m_{i}$, $r_{i}$, and $v_{i}$ are the quality, position vector, and velocity of particle *I*, and ${ƒ}_{i}$ is the total force that consists of three parts: conservative force $F_{ij}^{C}$, dissipative force $F_{ij}^{D}$, and random force $F_{ij}^{R}$.

(7) $${ƒ}_{i}=\underset{j\neq i}{\sum }\left(F_{ij}^{C}+F_{ij}^{D}+F_{ij}^{R}\right)$$

And the three forces can be given by:

$$F_{ij}^{C}=\left\{\begin{array}{c} -a_{ij}(1-|r_{ij}|){\hat{r}}_{ij},\;r_{ij}<r_{c}\\ \;\;\;\;\;\;\;\;\;\;\;\;\;\;\;0,\;r_{ij}≧r_{c} \end{array}\right.$$

(8) $$F_{ij}^{D}=\left\{\begin{array}{c} \lambda \omega^{D}\left(r_{ij}\right)\left({\hat{r}}_{ij}v_{ij}\right){\hat{r}}_{ij},\;r_{ij}<r_{c}\\ \;\;\;\;\;\;\;\;\;\;\;\;\;\;\;0,\;r_{ij}≧r_{c} \end{array}\right.$$

$$F_{ij}^{R}=\left\{\begin{array}{c} -\sigma \omega^{R}\left(r_{ij}\right){\hat{r}}_{ij}\varepsilon_{ij}\;\delta t^{-0.5},r_{ij}<r_{c}\\ \;\;\;\;\;\;\;\;\;\;\;\;\;\;\;0,\;r_{ij}≧r_{c} \end{array}\right.$$

where $r_{c}$ is the certain cutoff radius, which is set to 1 in DPD simulation system, and $r_{ij}=r_{i}-r_{j}$, ${\hat{r}}_{ij}=r_{ij}/\left|r_{ij}\right|$, $v_{ij}=v_{i}-v_{j}$**.**$\;a_{ij}$ is the maximum repulsion between two interacting beads. *ε_ij_* is a random number that averages to zero, indicating each interacting pair of beads is selected randomly at each time step δt.$\omega^{R},$ and $\omega^{D}$ are the weight functions for dissipative and random forces, respectively.

### 2.3. Model of DPD Simulation

Whether a model is reasonable or not plays a key role in the results of dissipative particle dynamics simulations. DPD simulation uses a series of beads to represent the atomic clusters in the system, which makes use of the flexible potential function to calculate the system energy and describes the movement of beads through the equation of motion and three kinds of forces [37]. Therefore, the polymer molecular chains need to be coarse-grained according to the formula below before conducting the DPD simulation. Therefore, it is crucial to construct suitable coarse-grained models for the NR/CR blends.

(9) $$\;NDPD=\frac{M_{p}}{M_{m}C\infty }=\frac{n}{C\infty }$$

where *N*_DPD_ is the number of beads in the molecular chain for the DPD simulation; $M_{p}$ is the molecular weight of the polymer; n is the polymerization degree of the polymer; and *C*_∞_ is the limiting characteristic ratio, which is an inherent property of the molecular chain.

**Figure 2:**
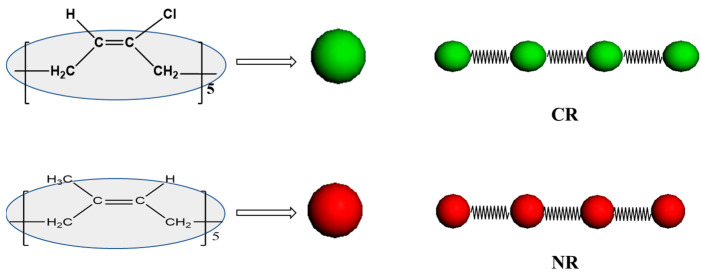
Scheme of NR and CR coarse-grained models. (Green corresponds to one CR molecule, and red corresponds to one NR molecule.)

The Synthia module of Materials Studio was used to calculate the limit characteristic ratio *C*_∞_ of NR and CR. The *C*_∞_ of NR and CR are found to be 5.45 and 5.44, respectively. As a consequence, the NR and CR coarse-grained models could be obtained by using 1 bead to replace 5 repeating units of NR and CR molecular chains, as shown in Figure 2. DPD accounts for the simulation at the mesoscopic scale, and the simulated system is much larger than that of molecular dynamics simulation. As a result, the molecular weight set during the simulation process is larger, and the specific coarse-grazing parameters in this work are shown in Table 2.

### 2.4. NR/CR Blending Experiments

The natural rubber/chloroprene rubber blends were prepared with a mechanical blending method. Table 3 shows the formulation of the NR/CR blends.

Firstly, NR was masticated in an open-roll mixing mill for 2 min, and then CR was added, and stearic acid, zinc oxide, promoter, sulfur, and other additives were gradually added according to the formula. The mixing process required repeated cutting knives operating, and the process was kept for 10 min. Secondly, the mixed compounds needed to be triangulated 4 times and crimped 4 times. Thirdly, the compounds were stored for 24 h to eliminate internal stress. Lastly, no rotor vulcanizer (MR-C3, Beijing Huanfeng Machinery Factory, Beijing, China) was used to test the vulcanization performance of the compounds, and then the compounds were vulcanized at 140 °C × 13.5 MPa on a platen vulcanizer to obtain the NR/CR blending vulcanizates.

A DMA test of the samples (width × thickness 4 mm × 1 mm, rectangular strips with suitable length) was carried out on a dynamic thermomechanical analyzer (Q850, TA Company, New Castle, DE, USA) in a film-stretching mode. The scanning temperature ranged from −130 °C to 60 °C, and the heating rate was 3 K/min. The frequency was 1 Hz, and the strain amplitude was 25 μm.

FT-IR spectra of the NR/CR blends were analyzed using a Fourier Transform Infrared Spectroscopy (T27, BRUKER, Billerica, Massachusetts, Germany). The specimens were scanned with attenuated total reflection (ATR) model in the range of 400–4000 cm^−1^.

## 3. Discussion and Results

### 3.1. Solubility Parameter and Flory–Huggins Parameter

Solubility parameter (δ) is an important parameter to characterize the polymer–solvent interaction and an important indicator to predict the miscibility between the dispersed phase and the continuous phase in a polymer blending system, which is defined as the square root of the cohesive energy density (CED):

(10) $$\delta =\sqrt{\mathrm{CED}}=\sqrt{\frac{E_{coh}}{V}}$$

where δ, $E_{coh}$, and *V* are solubility parameter, cohesive energy, and volume, respectively. For a molecular simulation, the more molecules and the longer chain length in the system, the more simulation time will be needed, and the higher the requirements of the calculation will be.

**Figure 3:**
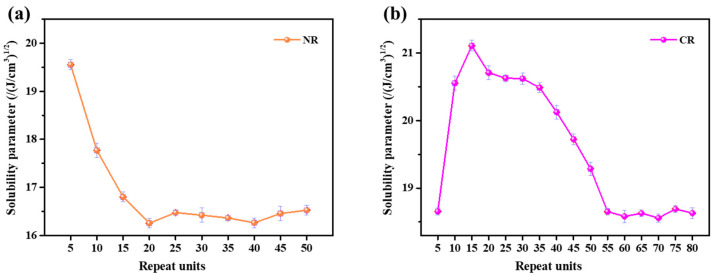
Relationship between repeat units and solubility parameter of (**a**) NR and (**b**) CR.

Figure 3 shows the relationship between repeating units and solubility parameter. As can be seen from Figure 3a, the solubility parameter of NR decreases with the increase of repeating units. When the number of repeating units reaches 20, the solubility parameter of NR tends to be constant. At the same time, as can be seen from Figure 3b, the solubility parameter of CR keeps stable when the repeating units reach 55. Thus 20 and 55 repeating units are sufficient to represent the NR and CR chain, respectively. In this study, 20 and 55 repeating units were selected as the molecular chain lengths of NR and CR, respectively, to construct the NR/CR blends model. The relative information of NR/CR blends model is shown in Table 4.

The Flory–Huggins interaction parameter χ is an important physical quantity for predicting the miscibility of a binary polymer blending system, which can be calculated as follows:

(11) $$\chi_{AB}=\left(\frac{\Delta E_{mix}}{RT}\right)V_{mono}$$

where *V_mono_* is the molar volume of the monomer, which can be calculated by dividing the total volume of the system by the total polymerization degree in the system. *R* and *T* are gas constant and temperature, respectively. $\Delta E_{mix}$ is the mixing energy blending system, which is given by:

(12) $$\Delta E_{mix}=\varphi_{A}{\left(\frac{E_{coh}}{V}\right)}_{A}+\varphi_{B}{\left(\frac{E_{coh}}{V}\right)}_{B}-{\left(\frac{E_{coh}}{V}\right)}_{mix}$$

where $\varphi_{A}$ and $\varphi_{B}$ represent the volume of component A and component B in the blending system. ${\left(\frac{E_{coh}}{V}\right)}_{A}$, ${\left(\frac{E_{coh}}{V}\right)}_{B}$, and ${\left(\frac{E_{coh}}{V}\right)}_{mix}$ are the cohesive energy density of component A, component B, and the blending system, which consists of A and B, respectively. According to the Flory–Huggins theory, the critical interaction parameter $\chi_{C}$ can be calculated with the following equation:

(13) $$\chi_{C}=\frac{1}{2}{\left(\frac{1}{\sqrt{n_{A}}}+\frac{1}{\sqrt{n_{B}}}\right)}^{2}$$

where $n_{A}$ and $n_{B}$ represent the minimum degree of polymerization of A and B.

The miscibility of the blends could be predicted by comparing the Flory–Huggins parameter and the critical interaction parameter of the two components in the blending system. If the Flory–Huggins parameter of the blends is bigger than the critical interaction parameter, it indicates that the blending system is miscible. In contrast, the blending system is immiscible.

**Figure 4:**
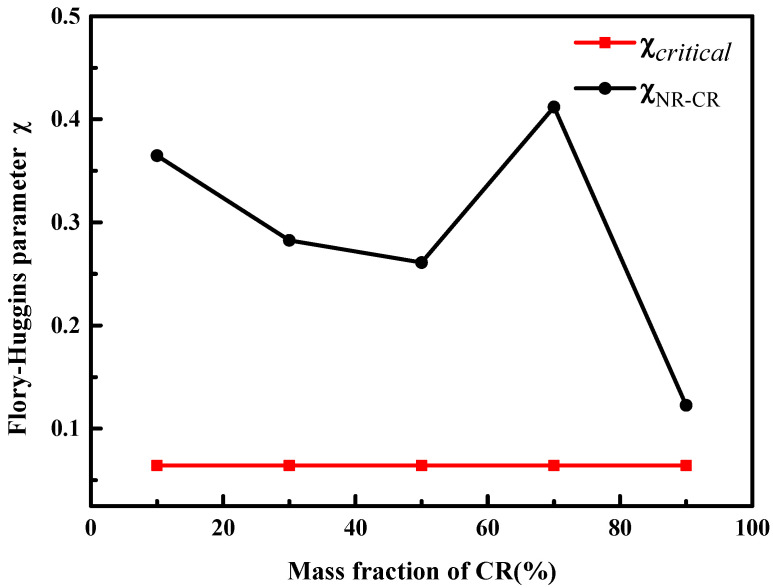
Flory–Huggins parameters of the NR/CR blends in different proportions. (Here, red line means the critical interaction parameter of NR/CR blends.)

The Flory–Huggins parameters of the NR/CR blends with different mass ratios are shown in Figure 4. As can be seen from Figure 4, the critical interaction parameter of NR/CR blends is 0.06. It can be found that the Flory–Huggins parameters between NR and CR are always higher than the critical interaction parameters regardless of the mass ratio of NR with CR, indicating that NR and CR are immiscible or partially miscible in all the blending systems. With the increase of CR in the blending system, the difference between the Flory–Huggins parameter and the critical interaction parameter seems to be reduced when the content of CR is less than 50%. In addition, the deviation between the Flory–Huggins parameter and the critical interaction parameter is the minimum when the content of CR is 90%.

### 3.2. Phase Diagrams of the NR/CR Blends

Phase diagram of polymer blends is an important method to study and predict the compatibility of polymer blends. It mainly contains three parts: critical point, spinodal curve, and binodal curve. These three sections divide the phase diagram into three regions. Inside the spinodal is the phase separation region, where the phase structure of the blend is unstable, and outside the binodal is the homogeneous region where the blend keeps stable. The region between the binodal curve and the spinodal curve is a metastable region, in which the blend is in a metastable state and easily affected by external conditions.

**Figure 5:**
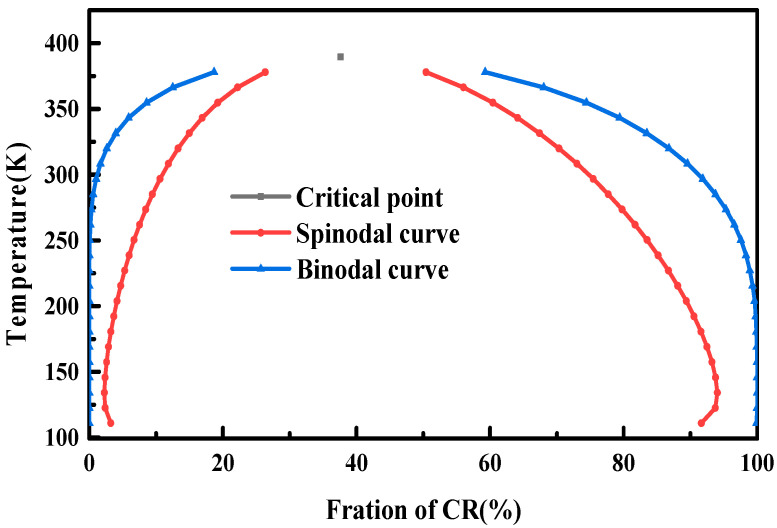
Phase diagram of the NR/CR blending system.

Figure 5 shows the phase diagram of the NR/CR blends. As can be seen from Figure 5, there is an upper critical temperature about 390 K in the NR/CR blends, which indicates that NR/CR blends are almost immiscible at room temperature. Only when the component of CR is close to zero or higher than 95%, NR and CR would be partially miscible. This conclusion is consistent with the research results of Saad and Ahn [38,39].

### 3.3. Density Variation along x-Axis of the Simulation Box

**Figure 6:**
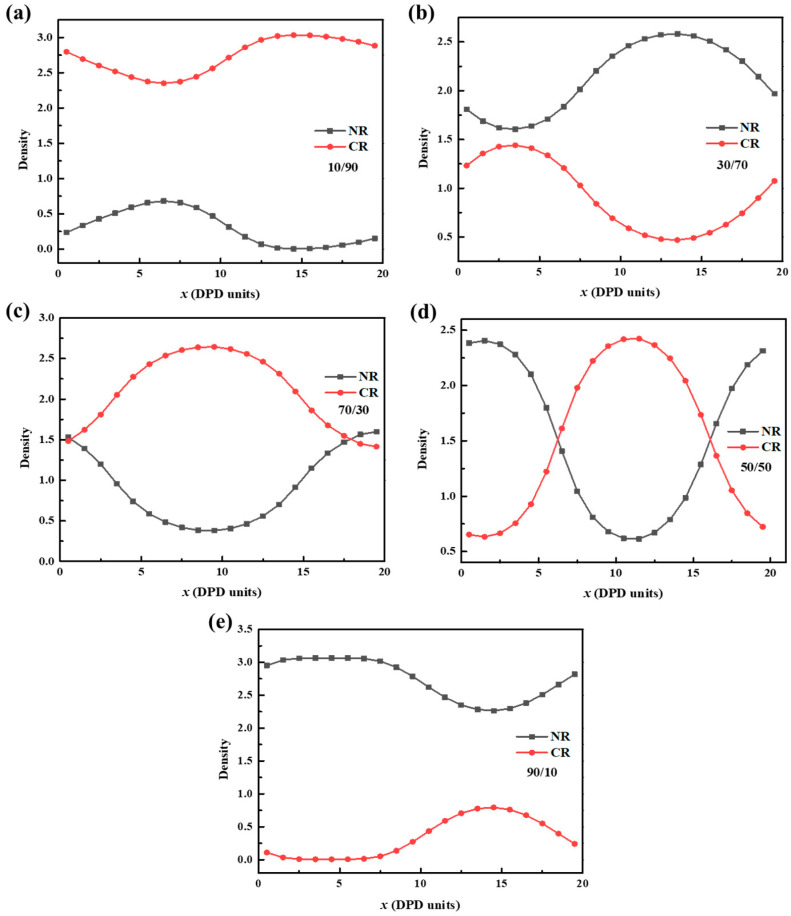
Variation of the density of different mass ratios (**a**) 10/90, (**b**) 30/70, (**c**) 50/50, (**d**) 70/30, and (**e**) 90/10 NR/CR blends along the *x*-axis of the simulation box.

The density variation of the NR/CR blends with different mass ratios along the *x*-axis of the simulated box was also obtained with the DPD simulation. It can be seen from Figure 6 that the density of the NR/CR blends at each ratio varies greatly along the X direction, and the values of the density variation are all above 0.5. When the NR/CR mass ratio is 10/90, the density of NR particles increases or decreases at the position of 0–15, indicating that NR aggregates within the boundary. For the sample with an NR/CR mass ratio of 30/70, the density of NR first decreases and then increases at the 3–20 position. The change trends of CR and NR are opposite, indicating that CR aggregates at this position. When the mass ratio is 50/50, the densities of NR and CR change as a mirror image. It shows that the NR and CR particles have spaced agglomeration at different positions along the *x* axis. Similarly, when the mass ratio is 70/30, the NR particles are enriched at 5–20 nm; when the mass ratio is 90/10, the CR particles are agglomerated at 7.5–20 nm. To sum up, the blending of NR and CR shows different degrees of aggregation of dispersed phases with different blending ratios, indicating that the compatibility of NR and CR is poor, and the blending of the two polymers will cause phase separation. Nevertheless, it can be found that microphase separation would take place rather than macroscopic phase separation. In this sense, the mechanical properties of the blends would not be impaired remarkedly. Meanwhile, design of the nanostructure could be achieved according to this finding.

### 3.4. Morphology of the NR/CR Blends

**Figure 7:**
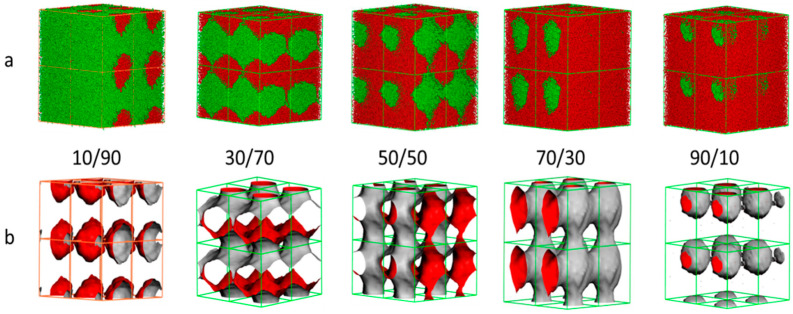
Morphologies (**a**) and iso-surface (**b**) of the NR/CR blends with different mass ratios. (In Figure 7a, red represents NR particles, and green represents CR particles. In Figure 7b, red represents the CR iso-surface, and gray represents the NR iso-surface.)

Figure 7 shows the morphologies of the NR/CR blends; Figure 7a shows the particles dispersion of the NR/CR blends (red represents NR particles, and green represents CR particles); and Figure 7b is the iso-surface maps of the NR/CR blends (red represents the CR iso-surface, and gray represents the NR iso-surface). From Figure 7a, it can be seen that there is obvious agglomeration in the NR/CR blends with any mass ratio. As can be seen from Figure 7b, there are different dispersed phase structures in different NR/CR blends. When the NR/CR ratio is 10/90 or 90/10, the spherical dispersed phase can be formed. While the mass ratio of NR with CR is 30/70, the spherical dispersed phase would be transformed to a lamellar structure. In addition, a bicontious structure can be found when the content of NR is 50% or 70%.

### 3.5. DMA Curves of the NR/CR Blends

**Figure 8:**
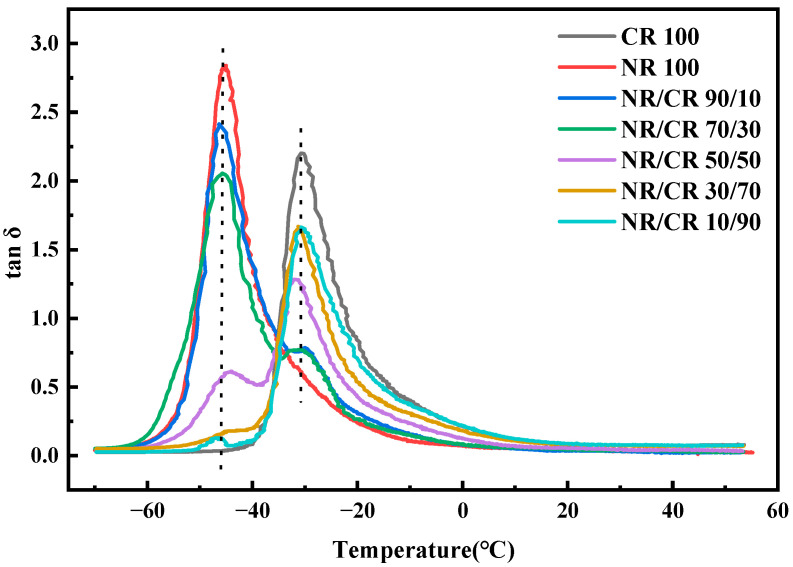
Curves of tan δ versus temperature for the NR/CR blends with different mass ratios.

Figure 8 shows the curves of tan δ versus temperature for the NR/CR blends with different mass ratios. It can be seen that the tan δ peaks of pure NR and CR can be found at 46 °C and 31 °C, respectively. In other words, the glass transition temperatures of NR and CR are 46 °C and 31 °C, respectively. The NR/CR blends with different mass ratios show two peaks, and the temperature of the two tan δ peaks are almost at the positions of the glass transition temperature of pure NR and pure CR. Therefore, there are two tan δ peaks corresponding to the glass transition temperature of pure NR and CR in the NR/CR blends, indicating that the NR/CR blends with different mass ratios have poor compatibility. Nevertheless, the two tan δ peaks shift to each other in some cases, which can be obviously observed with the incorporation of 50% or 90% CR in the blending system. The results of DMA agree well with the relationship between the Flory–Huggins parameter and critical interaction parameter in the NR/CR blends.

### 3.6. IR Curves of NR/CR Blends

**Figure 9:**
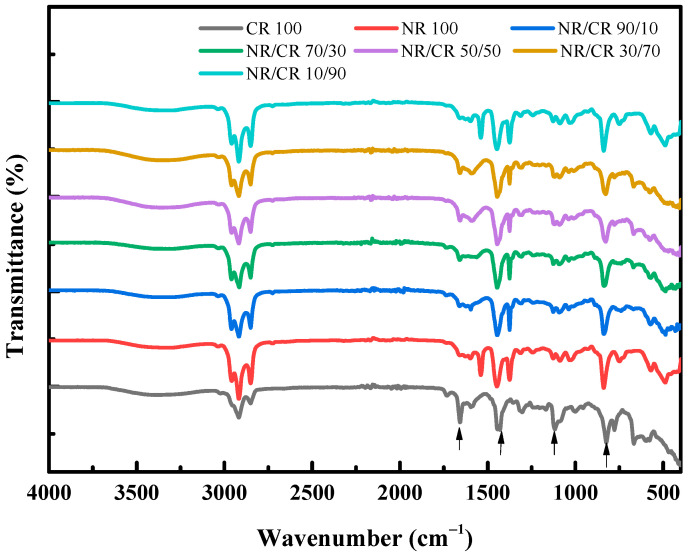
FT-IR curves of the NR/CR blends with different mass ratios.

Figure 9 shows the IR curve of the NR/CR blends. The black curve is the IR curve of CR, and the positions marked by the arrows are the characteristic bands of CR, which are 1667 cm^−1^ (stretching vibration of C=C), 1430 cm^−1^ (deformation vibration of methylene antisymmetric plane with chlorine atom in the adjacent position), 1117 cm^−1^ (vibration of carbon chain skeleton), and 824 cm^−1^ (corresponds to the C-H out-of-plane deformation vibration on the triple substitution C=C). The red curve is the NR IR curve, and the main characteristic band is 1375 cm^−1^ corresponding to the symmetric stretching vibration of methyl group, 838 cm^−1^ for the C-H out-of-plane deformation vibration of the cis-disubstituted carbon–carbon double bond, and 1450 cm^−1^ for the antisymmetric deformation vibration of methylene group.

It can be seen from Figure 8 that the IR curves of the NR/CR blends retain the characteristic bands of NR and CR without peak shifting or a new peak, indicating that the interaction between the two components of the NR/CR blends is weak, and the compatibility of the binary blends is poor.

## 4. Conclusions

In this work, molecular dynamics simulation and the DPD method were used to investigate the compatibility of the NR/CR blends system from interaction parameters, phase diagram, and morphologies of the blends. The results of the Flory–Huggins interaction parameter χ_NR-CR_ and the critical interaction parameter χ_c_ showed that the compatibility of the NR/CR blends with different mass ratios was poor. The maximum critical co-dissolution temperature of the NR/CR blends was calculated to be about 375 K according to phase diagram. The results of DPD simulation, FT-IR, and DMA also showed that the compatibility between NR and CR was poor. However, the miscibility could be improved when the content of CR was 50% or 90%. Furthermore, the diameters of the dispersed phase were calculated to be nanoscale according to the results of density variation along the *x*-axis of the simulation box. This work would be helpful for design of rubbery polymers with the guidance of molecular simulation.

## Figures and Tables

**Table 1:** Initial model-building parameters of the NR/BR blends.

System Number	NR/CR (Mass Ratio)	Number of Chain	Density (g/cm^3^)	Box Length (Å)
1	90/10	32NR/1CR	0.961	44.62
2	70/30	8NR/1CR	1.203	30.07
3	50/50	7NR/2BR	1.085	31.46
4	30/70	3NR/2BR	1.147	27.45
5	90/10	3NR/8NR	1.209	39.20

**Table 2:** Coarse-grained parameters of NR and CR.

Sample	Relative Molecular Weight	Limiting Characteristic Ratio	Particle Number
NR	300,000	5.45	810
CR	120,000	5.44	263

**Table 3:** Formulation of the NR/CR blends.

Sample Reagent	NR/CR 0/100	NR/CR 10/90	NR/CR 30/70	NR/CR 50/50	NR/CR 30/70	NR/CR 100/0
NR	0	10	30	50	30	100
CR	100	90	70	50	70	0
Stearic Acid	2	2	2	2	2	2
Zinc Oxide	5	5	5	5	5	5
Promoter CZ	0.5	0.5	0.5	0.5	0.5	0.5
Magnesium Oxide	5	4	3	2	1	0.5
Sulfur	0	0.5	1	1.5	2	2.5

**Table 4:** Parameters after geometric optimization of the NR/CR blending models.

NR/CR	10/90	30/70	50/50	70/30	90/10
$\varphi_{NR}$ (%)	12.39	36.22	56.98	75.31	92.37
$\varphi_{CR}$ (%)	87.61	63.78	43.02	24.69	7.63
CED_NR_ (×10^8^ J.m^−3^)	2.68	2.68	2.68	2.68	2.68
CED_CR_ (×10^8^ J.m^−3^)	3.50	3.50	3.50	3.50	3.50
CED_mix_ (×10^8^ J.m^−3^)	3.36	3.07	2.95	2.80	2.63
$\Delta E_{mix}$ (×10^6^ J.m^−3^)	4.19	13.9	8.62	9.20	11.7
*V*(Å^3^)	60,239.85	20,697.66	31,153.17	27,181.1	88,840.9
*V*_mono_ (×10^−5^ m^3^/mol)	7.26	7.33	7.50	7.61	7.69

## Data Availability

Not applicable.
